# Recurrent spontaneous hip dislocation in a patient with neurofibromatosis type 1: a case report

**DOI:** 10.1186/1752-1947-5-106

**Published:** 2011-03-16

**Authors:** John G Galbraith, Joseph S Butler, James A Harty

**Affiliations:** 1 Department of Trauma & Orthopaedic Surgery, Cork University Hospital & St. Mary's Orthopaedic Hospital, Cork, Ireland

## Abstract

**Introduction:**

Neurofibromatosis type-1 is a common genetic disorder which often affects the skeleton. Skeletal manifestations of neurofibromatosis type-1 include scoliosis, congenital pseudarthrosis of the tibia and intraosseous cystic lesions. Dislocation of the hip associated with neurofibromatosis type-1 is a rare occurrence and is underreported in the literature.

**Case presentation:**

We report a case of hip dislocation resulting from an intra-articular neurofibroma in an 18-year-old Caucasian woman following minor trauma. This was originally suggested by the abnormalities on early radiographs of her pelvis and later confirmed with computed tomography and magnetic resonance imaging. Treatment was successful with skeletal traction for six weeks with no further hip dislocations at a 12-year follow-up.

**Conclusion:**

This case illustrates the radiological features of this rare complication of neurofibromatosis type-1 using the modalities of plain radiograph, magnetic resonance imaging and computed tomography reconstruction. The radiological images give a clear insight into the mechanism by which neurofibromatosis type-1 leads to hip dislocation. It also demonstrates one treatment option with excellent results on long-term follow-up.

## Introduction

Neurofibromatosis type 1 (NF-1) is one of the most common autosomal dominant disorders affecting humans. It is estimated to affect 1 in 3,000 newborns and 1,000,000 people worldwide [1]. Friedrich Daniel von Recklinghausen, a German pathologist, was first to describe the neural involvement within affected tissues; hence, the association of his name with this disease. It is a disease involving tissues of ectodermal and mesoectodermal origin, particularly affecting skin, subcutaneous tissue, peripheral nerves and the skeleton.

The clinical features of NF-1 include: cafβ-au-lait spots, Lisch nodules, axillary freckling, optic gliomas and peripheral neurofibromas. NF-1 is a disease deeply relevant to orthopaedic surgery. Patients with NF-1 may present with characteristic orthopaedic manifestations such as scoliosis, congenital pseudoarthrosis of the tibia and limb hypertrophy[2]. Intraosseous cystic lesions, periosteal bone proliferation coxa valga and protrusion acetabuli have also been reported [3].

Dislocation of the hip associated with NF-1 is a rare occurrence. A comprehensive review of the literature revealed 12 cases of hip dislocation attributed to NF-1. We report a case of recurrent hip dislocation resulting from an intra-articular neurofibroma in an 18-year-old woman.

## Case report

An 18-year-old Caucasian woman with a history of NF-1 presented to the emergency department with pain in her left hip following minor trauma. She had tripped over her dog and landed on her left side. NF-1 had been diagnosed clinically in childhood. She had a histologically proven neurofibroma excised from her right forearm four years previously. She had a strong family history of NF-1, her mother and three second degree relatives exhibited clinical features.

**Figure 1:**
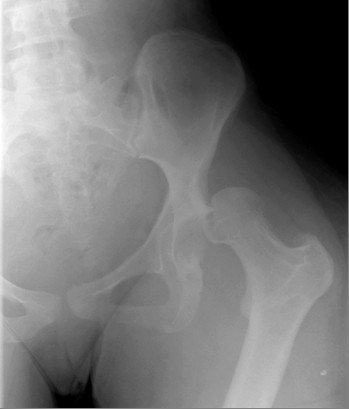
**Radiograph of dislocated left hip**.

On examination, we saw that her left leg was shortened, internally rotated, and adducted. There was decreased range of movement. She had diffuse swelling of her left lower limb with a distinct soft tissue mass above her right lateral malleolus. She had six cafβ-au-lait patches on her trunk and bilateral axillary freckling. A radiograph of the pelvis revealed a superior dislocation of her left hip with an abnormal appearing femoral neck (Figure 1).

Her hip was relocated under general anaesthetic and this was maintained with skin traction. A computed tomography (CT) of her pelvis displayed a smooth erosion of the lateral margin of her left ileum and femoral neck, markedly increased femoral neck offset, a concave abnormality superior to the left acetabulum and thinning of the left inferior pubic ramus. These changes, which appeared to be long standing, were accepted to be a result of a local neurofibroma causing bone erosion. Eight days post-operatively she experienced a sudden onset of left hip pain on attempting to move in bed. A radiograph of her hip revealed repeat dislocation. Relocation of her hip was performed under general anaesthetic and balanced skeletal traction was maintained by inserting a pin to her left proximal tibia. Skeletal traction was maintained for six weeks. She was mobilizing without aids at eight weeks and follow-up at three months revealed a normal hip examination.

**Figure 2:**
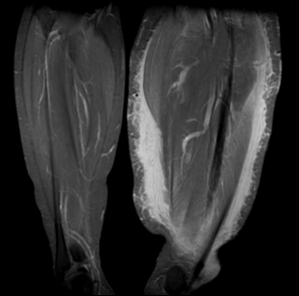
**Coronal MRI view of soft tissue swelling of left lower limb**.

**Figure 3:**
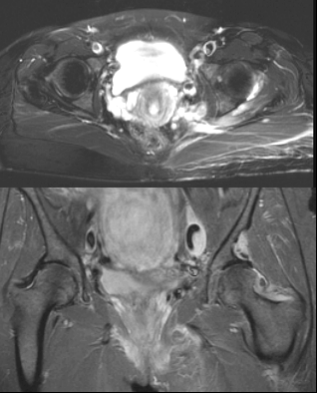
**Transverse and Coronal MRI views demonstrating neurofibroma above left femoral neck**.

**Figure 4:**
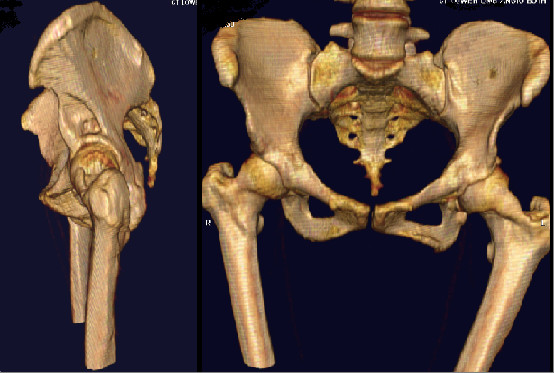
**CT reconstructions demonstrating erosion of left femoral neck and pelvis**.

**Figure 5:**
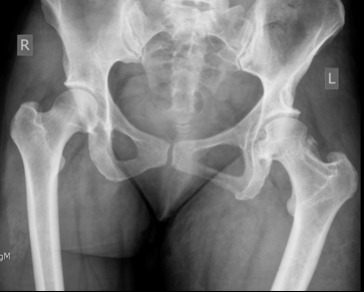
**Radiograph of pelvis demonstrate left hip to be in joint**.

She presented to the orthopaedic clinic six years later complaining of disfiguring hypertrophy of her left lower limb which had worsened markedly over the preceding two years. She was referred to plastic surgeons with a view toward performing a cosmetic debulking procedure. A pre-operative magnetic resonance imaging (MRI) scan of her lower limb demonstrated soft tissue swelling of her entire lower limb consistent with plexiform neurofibromatosis (Figure 2). An MRI of her pelvis displayed a 6 × 4 cm enhancing mass at the superior aspect of the left acetabulum extending into the left hip joint (Figure 3). There was smooth erosion of the neck of the femur but the head of the femur appeared to be in joint. An MRI of her lumbar spine was normal. A CT-guided biopsy of this hip lesion histologically confirmed it to be a neurofibroma. CT reconstructions demonstrated no further changes to the bone architecture of her left hip (Figure 4). In view of her lack of symptoms and the degree of operative difficulty expected, the peri-articular neurofibroma was not excised. She underwent several debulking procedures of her lower limb with excellent cosmetic results. At 12 years follow-up she has experienced no further dislocations of her left hip and mobilizes without aids with a normal gait. A radiograph of her pelvis demonstrated her left hip to be in joint (Figure 5).

## Discussion

Neurofibromatosis type-1 is a common genetic disorder that can have both focal and generalized skeletal manifestations. Generalized skeletal manifestations such as osteoporosis and short stature are common. Focal abnormalities such as tibial dysplasia, short angle scoliosis and sphenoid wing dysplasia, although less common, are well documented in the literature. However, there is a relative paucity of reported cases of pathological hip dislocation in patients with NF-1, with only 12 documented cases found in the published literature. Six dislocations occurred following trivial trauma [4-8]and six cases were deemed atraumatic [9-13].

The suggested mechanism for dislocation for the majority of cases has been related to the intra-articular growth of neurofibromas [4,5,8,10,13]. Local neurofibromas can lead to deformity of the pelvis, erosion of the neck of the femur, valgus deformity and joint capsule laxity, all of which predispose a person to dislocation. Neurofibromas distant from the hip joint have also been hypothesised to cause pathological dislocation. Mechanical instability due to weakness of the abductor muscles caused by spinal-cord tumors has been suggested as a predisposing factor [10]. Similarly, neurofibromas can lead to a deficiency of the normal sensation of the hip joint leading to a neuropathic arthropathy which can progress to dislocation [6].

Treatment described has ranged from conservative approaches to definitive surgical intervention, including Girdlestone resection [9] and total hip replacement [7]. Despite short-term good results from various treatments the long-term evidence is lacking, with no follow-up data longer than six years for any published case.

For our patient her hip dislocation was accepted to be a result of a neurofibroma impinging on the hip joint. This was originally suggested by the abnormalities on early radiographs of her pelvis and later confirmed with CT and MRI. The CT reconstructions of the hip area clearly demonstrate the mechanism by which hip dislocation has occurred (Figure 4). The smooth erosion of the lateral margin of the left ileum and femoral neck, the markedly increased femoral neck offset and the concave abnormality superior to the left acetabulum all contribute to the instability of the hip joint. The soft tissue swelling responsible for these abnormalities is clearly demonstrated on the MRI scan (Figure 3). There were no spinal-tumors displayed on the MRI of her lumbar spine. She was treated conservatively with skeletal traction. The short term results were excellent and at 12 years follow-up she has had no further episodes of dislocation.

## Conclusion

This case illustrates the radiological features of this rare complication of NF-1 using the modalities of plain radiograph, MRI and CT reconstruction. The radiological images give a clear insight into the mechanism by which NF-1 leads to hip dislocation. It also demonstrates one treatment option with excellent results on long-term follow-up.

## Consent

Written informed consent was obtained from the patient for publication of this case report and accompanying images. A copy of the written consent is available for review by the Editor-in-Chief of this journal.

## Competing interests

The authors declare that they have no competing interests.

## Authors' contributions

JG and JB collected data and drafted the manuscript. JH conceived the report. All authors critically appraised the manuscript and approved the final text.
